# The impact of mass distribution of long lasting insecticide-treated bed-nets on the malaria parasite burden in the Buea Health District in South-West Cameroon: a hospital based chart review of patient’s laboratory records

**DOI:** 10.1186/s13104-017-2870-8

**Published:** 2017-10-30

**Authors:** Renda Colins Yekabong, Walter Akoh Ebile, Peter Nde Fon, Emmanuel A. Asongalem

**Affiliations:** 1 Faculty of Health Sciences, University of Buea, Buea, Cameroon; 2 Department of Biomedical Sciences, Faculty of Sciences, University of Dschang, Dschang, Cameroon; 3 M.A SANTE (Meilleure Accès aux Soins de Santé), Yaoundé, Cameroon; 4 Solidarity Health Foundation/Solidarity Hospital Molyko, Buea, Cameroon

**Keywords:** Malaria, Hospital prevalence, Mean parasitaemia, Mass distribution, LLITN impact, Cameroon

## Abstract

**Background:**

Malaria remains a leading cause of illness and deaths in Cameroon. The use of long lasting insecticide treated bed nets (LLITN) is the most effective method to reduce the burden of malaria. The aim of this study was to determine the impact of the mass distribution of LLITN on the hospital prevalence of malaria (prevalence of malaria in patients with a presumptive diagnosis of malaria), in the Buea Health District in the South-West Region of Cameroon.

**Methods:**

A hospital-based chart review of records of malaria confirmatory test results in health facilities of the Buea Health District from January 2011 through December 2013. Data were extracted with the help of a grid, then analyzed with EPIinfo version 6 and Microsoft Excel 2010. Chi square test was used to compare prevalence and ANOVA was used to compare mean parasitaemia.

**Results:**

A total of 17,268 records were reviewed, 3545[20.5% (19.9–21.1)] confirmed malaria positive with mean trophozoite count of 2735.3 ± 23,323.5 trophozoite/µl of blood. Prevalence was higher in males 1497 [23.5% (22.4–24.5)] than females 2047 [18.8% (18.1–19.6)], *p* < 0.01. Significant evidence of a reduction in the prevalence of malaria was found in under-fives in 2012 (p = 0.03).

**Conclusions:**

Universal coverage with LLITN failed to guarantee effective control of malaria in the Buea Health District, as expected. Continuous and appropriate use of LLITN is indispensable, in addition to periodic sensitization, booster campaigns of LLITN distribution and evaluation research for effective prevention and control of malaria.

## Background

An estimated one million people in Africa die from malaria each year, most cases being children under 5 years and 90% of all deaths occurring in sub-Saharan Africa [1]. In Cameroon, malaria is endemic and a leading cause of illness and deaths. Malaria accounts for 31 and 44% cause of outpatient and inpatients consultations respectively [2], and about 14 and 54% of deaths in pregnant women and children under 5 years respectively [3]. The poorest people are at greatest risk. The direct and indirect costs associated with management of the disease pose substantial economic burdens to the families of the victims and the country at large [4].

Trends indicate that it is difficult, perhaps impossible for clinical management alone to control and prevent new cases and deaths associated with malaria. The government and her partners have made tremendous effort to improve access to basic health care that will especially benefit the poor. This includes provision of free treatment for simple malaria to children under five, free antenatal consultations and free intermittent preventive treatment of malaria for pregnant women in local health centers. Despite these efforts, malaria-associated morbidity and mortality rates are still unacceptably high. Among other reasons, this is mainly caused by varying levels of drug effectiveness, non-compliance to drug treatment, low or late turn-out in consultation units, and constant genetic variation of the plasmodium parasite.

In 2003, the government, through the national program on the fight against malaria, and her partners adopted a complementary strategy for the prevention and control of malaria. This involved the use of Insecticides Treated bed nets (ITN). Few years later, the program was expanded to the free mass distribution of LLITN in 2011. The use of LLITN is so far, the most effective way of reducing malaria morbidity and mortality, especially in children and pregnant women [5–8].

For an effective prevention and control of malaria, the achievement of universal coverage with LLITN is essential. To accomplish this, a nation-wide mass distribution of LLITNs was undertaken in December of 2011. However, little is known about the impact of this strategy (mass distribution of LLITN) on the burden of malaria parasite in Cameroon. In 2006 a community-based study assessed the impact of LLITN on malaria prevalence in a small locality in the South West Region of Cameroon [9]. However, information about the impact of LLITN on hospital prevalence of malaria and blood parasitaemia is lacking. The aim of this study was to assess the impact of the 2011 mass distribution of LLITN on the prevalence and the parasitaemia of malaria among patients with a presumptive diagnosis of malaria in the Buea Health District (BHD), the largest district in the South-West Region of Cameroon.

## Methods

### Study design

The study was a hospital-based chart review of malaria confirmatory test results (trophozoite per microliter of blood) in health facilities of the Buea Health District, recorded from January 2011 through December 2013. Data were extracted on a grid and analyzed in Epi Info version 6 and Microsoft Excel 2010.

### Study site

The BHD is the largest health district in the South-West Region of Cameroon. It shares boundaries with Mount Cameroon to the West and North, Muntegene to the South, and Ekona to the East. The district has a total of 27 health facilities that serve an estimated total population of 133,092 persons. These facilities include; one Regional Hospital, 9 public health centers, 2 missionary or confessional health facilities, 3 public infirmaries, one Para-public and 11 private health facilities. During the mass distribution of LLINs in 2011, the BHD recorded a percentage coverage of 91%, approximately 2.2 persons per bed net [10]. This is slightly above the World Health Organization’s (WHO) recommended value of one LLITN for every 1.8 people in the target population [11]. The study was conducted in the Buea Regional Hospital and the Muea Sub-Divisional Hospital. These two health facilities cover about 80% of the health needs of the entire population of the BHD, and are the only facilities that consistently recorded malaria test results in number of *trophozoite/µl* of blood as recommended by the WHO [12].

### Sampling

Only health facilities that recorded malaria test results in number of trophozoite/µl of blood were included. Those that recorded test results of malaria in number of pluses (++…) and malaria patients who did not do a confirmatory test were excluded. All the laboratory records for malaria confirmatory tests done from January 2011 through December 2013 in the selected health facilities were reviewed for data extraction.

### Data extraction process

In each health facility, all records covering the study period in the laboratory registers were reviewed. Subsequently, data were extracted and recorded chronologically with the help of a pre-tested grid. Each recorded result in a health facility was attributed a unique identification number for reference and to avoid double review. Variables for data extraction included the year test was done, the month, the age of patient, sex, and lab results in trophozoite/µl of blood. At the end of each day, the data grid was visually checked for missing data and duplicates. Data extraction was continuous for a duration of 45 days.

### Data management

The extracted data was then entered into a Microsoft Access database and exported into Epi Info version 6 and Microsoft Excel 2010 for analysis. All records that were reviewed and entered were considered in the analysis. The prevalence of malaria in the sample studied was calculated from the number of confirmed positive cases and the mean parasitaemia. The monthly and annual variation of the prevalence of malaria was determined through descriptive statistics, such as calculating proportions, and represented graphically. The impact of LLITN on the prevalence and parasitaemia of malaria was determined by comparing the prevalence of malaria and the mean parasitaemia over the 3 years span, using Chi square test for proportions and analysis of variance test (ANOVA) for means. The 95% confidence intervals of proportions were also computed. The results obtained for the year 2011 was the reference (pre-distribution results) used to assess the prevalence of malaria and mean parasitaemia in the following years (post-distribution). p value was used to determine the degree of impact that mass distribution of LLITN had on the prevalence of malaria and mean parasitaemia. A p < 0.05 indicates good evidence while a p < 0.01 indicates strong evidence.

## Results

### Characteristics of patients

A total of 17,268 records were reviewed in both health facilities (Buea Regional Hospital and Muea Sub-Divisional Hospital) over the study period. There were more female participants with a presumptive diagnosis of malaria 10,883 (63.0%) compared to males 6385 (37.0%). The mean age of the participants was 24.1 ± 18.9 years, while the most represented age groups were 22–40 years [n = 5959 (34.5%)] and those under-5 years [n = 3781 (21.9%)], summarized in Table 1.

**Table 1:** Characteristics of participants

Characteristics	BRH (%) N = 12,778 n (%)	MDH (%) N = 4490 n (%)	Total (%) N = 17,268
Sex			
Male	4564 (35.7)	1821 (59.4)	6385 (37.0)
Female	8214 (64.3)	2669 (64.5)	10,883 (63.0)
Age groups (years)			
0–5	2270 (17.8)	1511 (33.7)	3781 (21.9)
6–14	1382 (10.8)	752 (17.7)	2134 (12.4)
15–21	1839 (14.4)	576 (12.8)	2415 (14.0)
22–40	4838 (37.9)	1121 (25.0)	5959 (34.5)
> 40	2449 (19.2)	530 (11.8)	2979 (17.3)
Age (in years), mean ± SD	26.0 ± 18.8	18.7 ± 18.4	24.1 ± 18.9

### Hospital prevalence of malaria and mean parasitaemia

A total of 3545[20.5%, 95% CI 19.9–21.1] confirmed positive cases of malaria were recorded, identical to the prevalence of malaria in the Buea Health District during the study period. The overall mean trophozoite count was 2735.3 ± 23,323.5 trophozoite/µl of blood. There was very strong evidence that the prevalence was higher in males 1497 (23.5%) than in females 2047 (18.8%), p < 0.01. The highest prevalence was seen in the “school-age group” (6–14 years) 626 (29.3%) and children below 5 years 1075 (28.4%), as in Table 2. There was very strong evidence that the prevalence in Muea Sub-Divisional Hospital was greater than that in the Buea Regional Hospital (BRH) (p = 0.00).

**Table 2:** Hospital prevalence of malaria in the Buea Health district by age and sex (2011–2013)

	Population	# Positive cases	prevalence % (95% CI)	Mean parasitaemia T/µl ± (STD)
Age group (years)				
0–5	3781	1075	28.4 (27.0–29.9)	3668.7 ± 27,945.8
6–14	2134	626	29.3 (27.4–31.3)	5357.5 ± 41,819.8
15–21	2414	547	22.7 (21.0–24.4)	3125.3 ± 20,472.0
22–40	5956	911	15.3 (14.4–16.2)	1777.0 ± 14,953.4
> 40	2978	386	13.0 (11.8–14.2)	1269.5 ± 10,636.4
Sex				
Males	6383	1497	23.5% (22.4–24.5)	3520.0 ± 28,394.8
Females	10,880	2047	18.8% (18.1–19.6)	2273.4 ± 19,739.3
Health facility				
BRH	12,774	1714	13.4% (12.8–14.0)	3499.1 ± 27,029.0
MDH	4490	1831	40.8% (39.3–42.2)	558.5 ± 2569.1

In children under 5 years, the prevalence and mean parasitaemia dropped 1 year (2012) following distribution of LLITN then unexpectedly increased in 2013, as seen in Table 3.

**Table 3:** Hospital prevalence of malaria and parasitaemia in children under 5 years (2011–2013)

N = 3781	2011, N = 1142 n (%)	2012, N = 1154 n (%)	2013, N = 1485 n (%)
Prevalence	296 (25.9%)	254 (22.0)	525 (35.4)
Mean parasitaemia	4737.9 ± 39,154.3	3079.1 ± 21,798.1	3304.6 ± 20,978.1

**Fig. 1:**
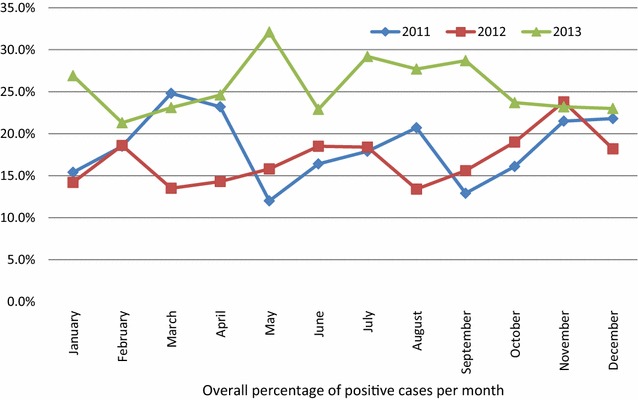
Monthly variation of malaria prevalence from 2011 to 2013

There was constant variation in the trend of malaria prevalence all year round. The highest and lowest malaria prevalences were both recoded in the month of May of 2013 and of 2011 respectively, as shown in Table 4 and Fig. 1.

**Table 4:** Annual and monthly variation of malaria and parasitaemia in the BHD from 2011 to 2013

Year	January	February	March	April	May	June	July	August	September	October	November	December	Total n.%
2011													
Total tested	465	351	363	526	507	463	447	368	521	547	548	577	5674
Positive (%)	70 (15.4)	65 (18.5)	90 (24.8)	122 (23.2)	61 (12.0)	76 (16.4)	80 (17.9)	76 (20.7)	67 (12.9)	88 (16.1)	118 (21.5)	126 (21.8)	1039 (18.3)
95% CI	[12.2, 19.1]	[14.7, 23.1]	[20.5, 29.6]	[19.7, 27.1]	[9.5, 15.3]	[13.2, 20.2]	[14.5, 21.8]	[16.7, 25.2]	[10.2, 16.1]	[13.2, 19.5,]	[18.2, 25.3]	[18.6, 25.5]	[17.3–19.3]
Mean parasitaemia (T/µl) ± STD	1358.7	6358.2	2021.4	3466.9	467.5	2027.3	1987.4	2662.2	2600.5	2562.2	4885.8	1956.4	2645.2 ± 25,240.0
2012													
Total Tested	479	307	458	414	474	428	495	471	455	479	526	329	5415
Positive (%)	68 (14.2)	57 (18.6)	62 (13.5)	59 (14.3)	75 (15.8)	79 (18.5)	91 (18.4)	63 (13.4)	71 (15.6)	110 (19.0)	125 (23.8)	60 (18.2)	920 (17.0)
95% CI	[11.3, 17.7]	[14.7, 23.1]	[10.6, 17.1]	[11.1, 18.1]	[12.7, 19.5]	[15.0, 22.5]	[15.1, 22.1]	[10.5, 16.9]	[12.5, 19.3]	[15.9, 22.5]	[20.2, 27.7]	[14.3, 22.9]	[16.0–18.0]
Mean parasitaemia (T/µl) ± STD	1352.5	1423.3	1093.7	1650	1758.8	4965.7	1763	1054.4	2779.6	2391.4	3183.6	3957.4	2257.2 ± 18,118.7
2013													
Total tested	513	450	428	574	657	476	449	419	561	602	574	470	6175
Positive (%)	138 (26.9)	96 (21.3)	99 (23.1)	141 (24.6)	211 (32.1)	109 (22.9)	131 (29.2)	116 (27.7)	161 (28.7)	143 (23.7)	133 (23.2)	108 (23.0)	1586 (25.7)
95% CI	[23.2, 31.0]	[17.7, 25.5]	[19.3, 27.5]	[21.1, 28.3]	[28.6, 35.9]	[19.3, 27.0]	[25.1, 33.7]	[23.5, 32.3]	[25.0, 32.7]	[20.4, 27.3]	[19.8, 26.9]	[19.3, 27.1]	[24.6–26.8]
Mean parasitaemia (T/µl) ± STD	4593.2	3027.4	3435.7	4258.4	3919.7	2318.6	2358	3879	3561.5	1480.9	3149.4	2725	3235.2 ± 25,441.2

### Impact of mass distribution of LLITN on the Hospital prevalence of malaria and parasitaemia

The prevalence of malaria in the study population in 2011 [n = 1039 (18.3%)] was compared with the prevalence in 2012 [n = 920 (17.0%)] and the prevalence in 2013 [n = 1586 (25.7%)]. There was not enough evidence that LLITN had reduced the prevalence of malaria in the general population in 2012 (p = 0.45) while there was strong evidence that the prevalence had increased in 2013 (p = 0.00). There was no significant evidence that the mean parasitaemia in the total sample had changed, when comparing 2012 and 2011, (p = 0.35) and between 2013 and 2011 (p = 0.21).

In the population of children under 5 years old, there significant evidence (p = 0.03) that the prevalence of malaria in 2012 [n = 254 (22.0%)] had dropped, compared to the prevalence in 2011 [n = 296 (25.9%)]. In 2013, very strong evidence indicated that the prevalence had increased instead [n = 525 (35.4%)] with p = 0.00. There was no evidence that the mean parasitaemia in children under five had reduced in 2012 or in 2013 when compared to 2011 (p = 0.15 and p = 0.23) respectively.

## Discussions

This was a hospital-based chart review of malaria confirmatory laboratory test results of patients, to determine the impact of mass distribution of LLITN on the hospital prevalence of malaria, and malaria parasitaemia in the BHD. Our study determined the prevalence of malaria at 20.5% (19.9–21.1) and the mean trophozoite count was 2735.3 ± 23,323.5 trophozoite/µl of blood. The most affected were; Children below 5 years (28.4%) and those in the “school-age group” (29.3%). We observed a constant variation in the monthly and yearly malaria prevalence and parasitaemia over the 3-year study period, as shown on Table 4 and on Fig. 1. We found that prevalence of malaria increased in 2013 compared to 2011 in the general population of study, while there was a decrease in malaria prevalence in children under five in 2012, and an increase in prevalence in 2013.

Despite the achievement of a universal coverage (91%) with LLITN in the BHD, the hospital prevalence of malaria and parasitaemia was still unacceptably high. This prevalence was twice as high as the national prevalence in 2013 [13]. A community based study conducted in 2006 showed about a twofold higher prevalence [9], than the prevalence obtained in this study. The difference in values of the prevalence obtained in the community-based study compared to this study could possibly be explained by the absence of free mass distribution of LLITN in 2006, and by the difference in study methods (community based and review of hospital records). In addition, from 2006 to 2013, there was an increase in the intermittent preventive treatment and prenatal consultations, as well as general community education on malaria preventive measure. Other studies in different regions of the country have obtained different prevalence values [14, 15] but these studies were house hold surveys and based their findings on the results obtained from rapid diagnostic test, recorded as ++…., This observation supports the fact that malaria prevalence varies significantly from one eco-geographical zone to another across the country [16]. However, our study had similar results to a community based study in a locality in Nigeria [17].

Our study demonstrated that malaria prevalence varies significantly with regards to time, age, and gender. We observed constant fluctuations in prevalence from 1 month to another (Fig. 1) and between the years, with a much higher prevalence in 2013 compared to each of 2011 and 2012. The highest prevalence in 2013 occurred in May, in March for 2011 and in November for 2012. These are periods of the year that mark the start, middle and the end of the rainy seasons. We also observe the lowest prevalence in the months of May for 2011, August for 2012 and February for 2013. In this study, the seasonal variation in the prevalence of malaria in the BHD demonstrated a significantly difference from the trends demonstrated in other studies across the country [18–20]. This can be explained by the fact that the other studies were conducted prior to the mass distribution of LLITN. Thus, the seasonal pattern in prevalence recorded in this study may have been influenced by the presence of the LLITN, given that mass distribution was in the month of December 2011.

Prevalence and parasitaemia were significantly higher in males than in females (p < 0.01). Several studies in other sub-Saharan African countries have shown similar results [18–20]. This is mostly because women are more likely to use bed nets than males [21] and some women (pregnant) also benefit from the free intermittent preventive treatment offered in health facilities. The most affected age groups were “school-age group” (6–14 years) and children below 5 years old. This is in line with the finding that those in the “school-age group” are the least likely group to use LLITN [21, 22]. This additionally explains why the prevalence was higher in this group than in children below five [23] who are generally followed up by their mothers to ensure that they sleep under bed nets.

There was enough evidence to suggest that the mass distribution of LLITN contributed to reducing the hospital-based prevalence of malaria in children under 5 years in 2012, just about a year following the implementation of the program. This supports the results of other studies that have demonstrated that the proper use of LLITN significantly reduces malaria morbidity and mortality [9, 24–26]. There was also a reduction in the prevalence of malaria in the total population reviewed, 1 year after the implementation of the mass distribution of LLITN program. However, we observed an unexpected increase in the prevalence of malaria in both the general population and in children under 5 years during the second year following the mass distribution of LLITN. The mean parasitaemia had changed in both general population and in children below 5 years old, for both the first and second year. The regular and proper use of LLITN has been proven to have significant impact in the reduction of malaria transmission and malaria burden [8]. This boosts the fact that, in spite achieving a universal coverage with mass distribution of LLITN, the regular and proper use is indispensable for an effective prevention and control. From our results, we believe that the regular and correct use of LLITN by the population was effective within the first few months after receiving the bed nets. However, it is likely that the rate of regular and correct usage reduced significantly over time. In addition, the distribution of LLITNs is supposed to be followed-up with drills of proper mounting of the nets and post distribution campaigns to encourage the population to use the nets effectively. These drills were lacking in this campaign. These may explain why there was an unexpected increase in the prevalence of malaria 2 years following distribution of LLITN. This is supported by results of a study that demonstrated that the rate of use of LLITN by populations significantly reduces with time, far below the levels required to create significant impact on the burden of malaria [27].

### Limitations

Test results relied on the technical capacity of the laboratory personnel to identify and count trophozoite and document correct test results. We did not assess their experience and technical capacity. Secondly, only patients with presumptive diagnosis of malaria, who were referred for laboratory confirmation, were included. Those who were asymptomatic and those who had self-treatment for malarial symptoms were not included in the study. Thirdly, some health facilities in the district were excluded because they failed to quantify the parasitaemia, for example, by recording results as number of “pluses” (++…), following the use of the rapid diagnostic test method. This is not the recommended method of testing. However, these health facilities cover only a small percentage of the BHD population. Therefore, the results obtained in this study are not a perfect representation of the community-based prevalence in the entire Buea Health District and should be interpreted with the above considerations.

## Conclusion

Malaria remains a serious public health problem in the Buea Health District with high prevalence and high parasitaemia. The prevalence studies showed an inconsistent pattern throughout the years, unlike other studies. The most affected populations groups were children under five, “school-age group” and males. We found that the mass distribution of LLITN did not reduced the burden of malaria in the BHD as expected. Rather, there was a seemingly increase in the prevalence and mean parasitaemia in patients with a presumptive diagnosis of malaria over time compared to the initiation of mass distribution of LLITN. Given that we observed an immediate (short-term) positive impact of the distribution of LLITN, we believe this was due to regular and correct use in the first few months after reception of the LLITN. With time, as the frequency of usage dropped, it resulted in an increase in malaria prevalence and parasitaemia. This suggested that the simple provision of LLITN to local populations is not a guarantee for a lasting decrease in the burden of malaria. Regular, continuous, and appropriate use is indispensable to guarantee an effective prevention and control of malaria. Hence, to succeed in the reduction of malaria-associated morbidity and mortality through the mass distribution of LLITN, we recommend periodic post-distribution community sensitization campaigns on the use of LLITN, to ensure sustainability and booster distribution campaigns. In addition, students, and those in the “school-age group” should be educated in school on the methods of prevention of malaria and the importance of using constant and appropriate use of LLITN, given that they are the most affected age groups. Finally, there should be intensified health education and sensitization programs targeting especially mothers, who are generally the caretakers of their under-five children.

## Abbreviations

BHD: Buea Health District
CI: confidence interval
p value: evidence of association
T/µl: trophozoites per microliter
STD: standard deviation
Yrs: years
LLITN: long lasting insecticide treated bed nets
MDH: Muea Sub-Divisional Hospital
BRH: Buea Regional Hospital
WHO: World Health Organization

## References

[1] World Health Organization: World Malaria Report. 2014. http://www.who.int/malaria/publications/world_malaria_report_2014/wmr-2014no-profiles.pdf. Accessed 17 Sept 2015.

[2] Songue E, Tagne C, Claudel M, Pretty E, Paul S, Roger M (2013). Epidemiology of malaria in three geo-ecological zones along the Chad-Cameroon pipeline. Am J Epidemiol Infect Dis..

[3] Ndong B, Fondjo E, Kouambeng C, Zambou B, Massoda T, Achu F (2009). The situation of malaria contro in 2008.

[4] Okorosobo T, Fola O, Germano M, Juliet NO, Joses MK (2011). Economic burden of malaria in six Countries of Africa. Eur J Bus Manag..

[5] Takken W (2002). Do insecticide-treated bed nets have an effect on malaria vectors?. Trop Med Int Health.

[6] Jones G, Steketee RW, Black RE, Bhutta ZA, Morris SS (2003). How many child deaths can we prevent this year?. Lancet..

[7] Phillips-Howard PA, Nahlen BL, Kolczak MS, Hightower AW, Kuile FOT, Alaii JA (2003). Efficacy of Permethrin-treated bed nets in the prevention of mortality in young children in an area of high perennial malaria transmission in Western Kenya. Am J Trop Med Hyg.

[8] Lengeler C (2004). Insecticide-treated bed nets and curtains for malaria control. Bull World Health Organ..

[9] Nkuo-Akenji T, Ntonifor NN, Ndukum MB, Kimbi HK, Abongwa EL, Nkwescheu A (2006). Environmental factors affecting malaria parasite prevalence in rural Bolifamba, South West Cameroon. Afr J Health Sci.

[10] South West Regional Health Service Report, 2012.

[11] World Malaria Report. Report. 2010 2010. Report No.

[12] Killeen GF, Chitnis N, Moore SJ, Okumu FJ (2011). Target product profile choices for intra-domiciliary malaria vector control pesticide products: repel or kill?. Malaria J.

[13] National Institute of Statistics. Cameroon Annual Statistics. 2013. http://www.statistics-cameroon.org/news.php?id=170.

[14] Songue E, Tagne C, Mbouyap P, Essomba P, Moyou Somo R (2013). Epidemiology of malaria in three geo-ecological zones along the Chad-Cameroon pipeline. Am J Epidemol Infect Dis..

[15] Akenji TN, Ntonifor NN, Kimbi HK, Abongwa EL, Ching JK, Ndukum MB (2005). The epidemiology of malaria in Bolifamba, a rural community on the eastern slopes of Mount Cameroon: seasonal variation in the parasitological indices of transmission. Ann Trop Med Parasitol.

[16] World Health Organization (1980). Manual of basic techniques for a health laboratory.

[17] Abdullahi K, Adamu U, Adamu T, Daneji AI, Aliyu RU, Jiya N (2009). Malaria in Sokoto, North West Nigeria. Afr J Biotech.

[18] Yeshiwondim AK, Gopal S, Hailemariam AT, Dengela DO, Patel HP (2009). Spatial analysis of malaria incidence at the village level in areas with unstable transmission in Ethiopia. Int J Health Geogr..

[19] Baume CA, Marin MC (2007). Intra-household mosquito net use in Ethiopia, Ghana, Mali, Nigeria, Senegal and Zambia: are nets being used? Who in the household uses them?. Am J Trop Med Hyg.

[20] Baume CA, Reithinger R, Woldehanna S (2009). Factors associated with use and non-use of mosquito nets owned in Oromia and Amhara Regional states, Ethiopia. Malaria J..

[21] Garley AE, Ivanovich E, Eckert E, Negroustoueva S, Ye Y (2013). Gender differences in the use of insecticide-treated nets after a universal free distribution campaign in Kano State, Nigeria: post-campaign survey results. Malaria J..

[22] Noor AM, Kirui VC, Brooker SJ, Snow RW (2009). The use of insecticide treated bed nets by age: implications for universal coverage in Africa. BMC Public Health..

[23] Christian M. Factors influencing mosquito bed net use in households in the Buea Health District. University of Buea; 2012.

[24] Bhattarai A, Ali AS, Kachur SP, Martensson A, Abbas A, Khatib R (2007). Impact of Artemisinin-based combination yherapy and insecticide treated nets on malaria burden in Zamzibar. PLoS Med..

[25] Kyu HH, Georgiades K, Shannon HS, Boyle MH (2013). Evaluation of the association between long-lasting insecticidal nets mass distribution campaigns and child malaria in Nigeria. Malaria J..

[26] Binka FN, Indome F, Smith T (1998). Impact of spatial distribution of permethrin-impregnated bed nets on child mortality in rural Northern Ghana. Am J Trop Med Hyg..

[27] Alliance for Malaria Prevention. Givewell, 2011.

